# Upregulation of HLA Class I Expression on Tumor Cells by the Anti-EGFR Antibody Nimotuzumab

**DOI:** 10.3389/fphar.2017.00595

**Published:** 2017-10-06

**Authors:** Greta Garrido, Ailem Rabasa, Cristina Garrido, Lisset Chao, Federico Garrido, Ángel M. García-Lora, Belinda Sánchez-Ramírez

**Affiliations:** ^1^Tumor Immunology Direction, Molecular Immunology Institute, Center of Molecular Immunology, Havana, Cuba; ^2^Servicio de Análisis Clínicos e Inmunología, Hospital Universitario Virgen de las Nieves, Granada, Spain; ^3^Departamento de Bioquímica, Biología Molecular e Inmunología III, Universidad de Granada, Granada, Spain

**Keywords:** nimotuzumab, EGFR, HLA class I molecules, antibodies, tumor cells

## Abstract

Defining how epidermal growth factor receptor (EGFR)-targeting therapies influence the immune response is essential to increase their clinical efficacy. A growing emphasis is being placed on immune regulator genes that govern tumor – T cell interactions. Previous studies showed an increase in HLA class I cell surface expression in tumor cell lines treated with anti-EGFR agents. In particular, earlier studies of the anti-EGFR blocking antibody cetuximab, have suggested that increased tumor expression of HLA class I is associated with positive clinical response. We investigated the effect of another commercially available anti-EGFR antibody nimotuzumab on HLA class I expression in tumor cell lines. We observed, for the first time, that nimotuzumab increases HLA class I expression and its effect is associated with a coordinated increase in mRNA levels of the principal antigen processing and presentation components. Moreover, using 7A7 (a specific surrogate antibody against murine EGFR), we obtained results suggesting the importance of the increased MHC-I expression induced by EGFR-targeted therapies display higher in antitumor immune response. 7A7 therapy induced upregulation of tumor MHC-I expression *in vivo* and tumors treated with this antibody display higher susceptibility to CD8^+^ T cells-mediated lysis. Our results represent the first evidence suggesting the importance of the adaptive immunity in nimotuzumab-mediated antitumor activity. More experiments should be conducted in order to elucidate the relevance of this mechanism in cancer patients. This novel immune-related antitumor mechanism mediated by nimotuzumab opens new perspectives for its combination with various immunotherapeutic agents and cancer vaccines.

## Introduction

The molecular understanding of tumor biology has advanced significantly over the past two decades. Multiple studies have discovered crucial pathways that drive tumor growth and progression, generating carefully designed drugs that can attenuate these circuits. Interestingly, several of these molecular routes are also essential to support the communication between the tumor and its microenvironment, including the infiltrating immune cells. This idea raises the possibility that some components of the antitumor efficacy of targeted therapies might involve the participation of the host immune system. EGFR signaling is one of the best-characterized pathways that promote the development of epithelial tumors (Yarden and Pines, 2012). EGFR was proposed as a target for cancer therapy nearly 30 years ago, and currently, monoclonal antibodies (mAbs) and tyrosine kinase inhibitors (TKIs) targeting EGFR are commercially available for cancer treatment (Tebbutt et al., 2013). Although, EGFR-inhibitors have had clinical success there is a substantial room for progress in improving their clinical efficacy. For example, the complete elucidation of the mechanisms of action for EGFR-inhibitors (EGFRIs) is still pending. EGFR-targeting agents were initially developed to attenuate the oncogenic signaling (that is, intrinsic pro-tumoral functions) (Seshacharyulu et al., 2012). However, there is growing evidence that EGFRIs could also affects the branches of the immune system, especially the adaptive immunity. Recent data have shown that EGFR-specific CD8^+^ T cells may contribute to clinical response in cancer patients treated with anti-EGFR mAbs (Srivastava et al., 2013). In addition, some mAbs and TKIs now used in patients have been validated as positive regulators of the tumor expression of HLA class I and antigen processing machinery (APM) components (Kersh et al., 2016).

Nimotuzumab, also known as h-R3, is a humanized anti-EGFR mAb. Clinical trials and therapeutic use of this antibody, involving > 40000 patients worldwide, have shown clinical efficacy in patients with advanced epithelial-derived tumors (Cabanas et al., 2013; Massimino et al., 2014; Reddy et al., 2014; Zhai et al., 2015). Compared with other anti-EGFR therapies the low toxicity and the lack of skin rash from nimotuzumab is an advantage (Crombet et al., 2004; Garrido et al., 2011b; Bode et al., 2012; Reddy et al., 2014). At present, nimotuzumab is approved for therapeutic use in cancer treatment in many countries (Ramakrishnan et al., 2009; Reddy et al., 2014). The clinical effect of nimotuzumab has been observed when the drug was used alone or in combination with radiotherapy, chemotherapy or chemoradiation (Massimino et al., 2014; Wang et al., 2015; Huang et al., 2017). The underlying molecular mechanism of action of nimotuzumab is believed to be associated with inhibition of the EGFR signaling and subsequently the related oncogenic events (Crombet-Ramos et al., 2002). However, its extrinsic anti-tumoral functions have not been extensively explored. To investigate the possible immune effect, we studied the regulation of HLA class I and APM components expression by nimotuzumab in human tumor cell lines. Moreover, we used a surrogate antibody that recognizes murine EGFR named 7A7 (Garrido et al., 2007, 2011a) to simulate the effect of nimotuzumab *in vivo* using immunocompetent mice.

## Materials and Methods

### Cell Lines and Reagents

Mouse tumor models studied were D122 metastatic clone of the Lewis lung carcinoma (Eisenbach et al., 1984), F3II mammary adenocarcinoma (Alonso et al., 1998), CT26 colon carcinoma (ATCC CRL-2638), 4T1 mammary carcinoma (ATCC CRL-2539), and B16F10 metastatic clone of B16 melanoma (Poste et al., 1980). For human tumor models we used A431 (epidermoid carcinoma; ATCC CRL-1555), H125 (lung adenocarcinoma) (Carney et al., 1985), and U1906 (small cell lung carcinoma) (Bergh et al., 1985) cell lines. Cells were grown in RPMI-1640 medium supplemented with 10% fetal calf serum (FCS) and antibiotics (Life Technologies, Gaithersburg, MD, United States). All cells were grown at 37°C in a humidified 5% CO_2_ incubator. 7A7 and nimotuzumab were obtained at the Center of Molecular Immunology (Havana, Cuba). Cetuximab was manufactured by Merck Pharma GmbH (Deutschland, Germany). AG1478 was purchased from LC Laboratories (Woburn, MA, United States). AG1478 was dissolved in DMSO for *in vitro* studies and in 0.05% (v/v) Tween 80 solution for *in vivo* use. Mouse and human IFN-γ were purchased from Sigma (St. Louis, MO, United States). Mouse EGF that stimulates efficiently mouse and human EGFR-positive cells was obtained from R&D Systems (Minneapolis, MN, United States). Antibodies specific for H-2K^b^ (HB-11), H-2D^b^ (HB-27), H-2K^d^ (K9-18), H-2D^d^ (34-5-8), and HLA-ABC (W6/32) molecules were obtained from ATCC mouse hybridomas (Rockville, MD, United States) and used for flow cytometry analysis. Antibodies specific for HLA-A (108265) (Lozano et al., 1989) and HLA-B (42-IB5) (Lozano et al., 1990) molecules were also used for flow cytometry analysis.

### *In Vitro* Treatment of Tumor Cells

Cells were plated (1.25 × 10^5^) in 10% FCS RPMI-1640 medium in six-well plates (Costar, Cambridge, MA, United States). To measure the effect of EGFR activation on MHC-I HC, β_2_-m and APM components expression, 12 h later the tissue culture medium was replaced with 1% FCS RPMI-1640 containing EGF (0.5 nM for mouse cells or 0.08 nM for human cells) and cells were incubated for 48 h. Control cells were cultured in absence of EGF. To determine the effect of EGFRIs on MHC-I HC, β_2_-m and APM components expression, 12 h later AG1478 (5 μM), 7A7 (1 μg/ml), nimotuzumab or cetuximab (10 μg/ml) were added in 1% FCS RPMI-1640 medium supplemented with EGF and the cells were incubated for an additional 12, 24, 48, 72, 96, or 120 h. Control cells were grown without EGFRI treatment. Maximum induction of MHC-I expression was obtained by treatment with mouse (100 UI/ml) or human (800 UI/ml) IFN-γ during 48 h.

### Flow Cytometry Studies

EGFR membrane expression was analyzed in mouse cell lines using 7A7 as primary antibody. MHC-I membrane expression was detected in mouse and human cells in basal conditions and after indicated treatments using specific antibodies described above. FITC-labeled rabbit anti-mouse Ig (Fab_2_) (Sigma) was used as a secondary antibody. Flow cytometry experiments were done according to standard methods: 5 × 10^5^ cells were washed twice in PBS supplemented with 2% FCS and 0.1% sodium azide. Cells were incubated with the primary antibodies at saturating concentration for 30 min at 4°C. The secondary antibody was used at a 1:100 dilution and incubated with cells for 30 min at 4°C in the dark. Isotype-matched non-immune mouse IgG and cells labeled with only the FITC-conjugated antibody were used as controls. Analyses were performed on a BD FACScan flow cytometer and the data were processed using WinMDI 2.8 software package (The Scripps Research Institute, La Jolla, CA, United States).

### *In Vivo* Tumor Study

All animal studies were carried out in accordance with the recommendations of the Institutional Animal Care and Use Committee of the Center of Molecular Immunology which approved the protocols. Six- to eight-week-old female C57BL/6 mice (Center for Laboratory Animal Production, Havana, Cuba) were challenged with D122 cells (5 × 10^5^) subcutaneously (s.c.) into dorso-lumbar regions and the development of primary tumors was monitored. The largest perpendicular diameters of the resulting tumors were measured with caliper and tumor volume was calculating using the formula: π/6 × length × width^2^. Twelve days later when tumor reached ∼0.25 cm^3^, the animals were randomly separated into six groups: intratumoral 7A7 (56 μg), intratumoral AG1478 (1 mg), intratumoral control (PBS), systemic 7A7 [56 μg intraperitoneal (i.p.)], AG1478 systemic (1 mg orally) and systemic control (PBS i.p.). EGFRIs administration began at day 12 and continued every 72 h until day 21. On day 22 mice were euthanized and primary tumors were removed, digested and prepared as a single-cell suspension. MHC-I surface expression was analyzed in cell suspensions by flow cytometry as described above.

### Cytotoxicity and IFN-γ ELISA Assays

D122-specific CD8^+^ T cells were generated as described previously (Garrido et al., 2011a) and co-cultured with D122 or B16F10 cells pretreated with EGFRIs (96 h) or IFN-γ (48 h). Cytotoxic activity was determined in 4 h using an *in vitro* lactate dehydrogenase assay. Percentage of specific lysis was calculated as: ([experimental release – effector cell release – spontaneous release]/[maximum release – spontaneous release]) × 100. Maximum release was obtained by adding 1% Triton X-100 to target cells, and spontaneous release was determined by incubating target cells with medium alone. Culture supernatants were collected after 48 h and IFN-γ concentration was determined by mouse IFN-γ ELISA kit (eBioscience, San Diego, CA, United States) according to manufacturer’s instructions. To measure the relative contribution of H-2K^b^ versus H-2D^b^ alleles to antitumor CD8^+^ T cell response, antibodies specific for these molecules (10 μg/ml) were added during the 48 h incubation period.

### RNA Extraction and Real-Time RT-PCR Analysis

Total RNA from cells was isolated by TRIzol method and was reverse transcribed using a High Capacity Reverse Transcription Kit (Applied Biosystems, Foster City, CA, United States). SYBR Green-based quantitative real-time PCR for several mouse and human genes was performed with the Applied Biosystem 7500 Real Time PCR System. Samples for each experimental condition were run in quadruplicate using mouse or human GADPH as housekeeping genes. PCR conditions were 40 cycles of 15 s of denaturation at 95°C and 60 s at 60°C. Primer sequences were manually designed and listed in Supplementary Table S1. The expression of each target gene is presented as the fold change relative to the expression in untreated cells.

### Statistical Analysis

Data were expressed as means ± SD. The Student’s *t*-test was used to compare mean values. For comparisons between three or more groups, an analysis of variance (ANOVA) was performed, followed by multiple comparisons of means by Dunnet or Bonferroni post-test. When the variables were not normally distributed, Mann–Whitney *U*-test was used to compare variables between two groups. When three or more groups were compared, the Kruskall–Wallis test with Dunn post-test was used. A significance level of *P* < 0.05 was assumed for all statistical tests. SPSS software (IBM, Armonk, NY, United States) was used for the data analyses. All statistical tests were two-sided.

## Results

### Nimotuzumab Augments HLA Class I Cell Surface Expression in Tumors via a Coordinated Transcriptional Increase for Several Antigen Processing and Presentation Machinery Components

**FIGURE 1:**
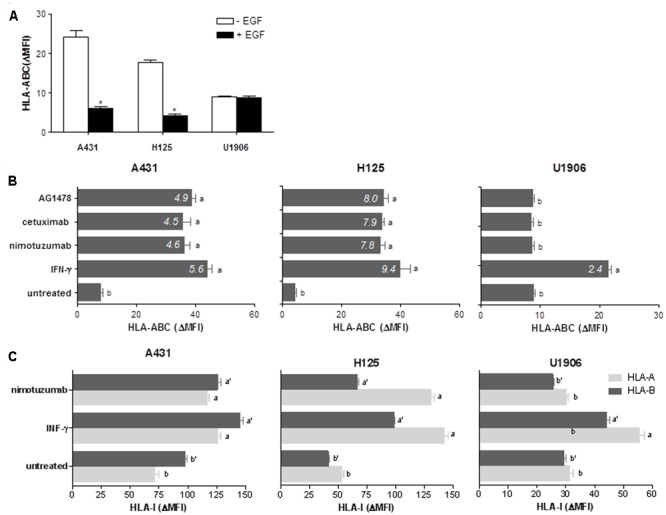
Nimotuzumab-induced EGFR inhibition enhances surface expression of HLA-I antigens in human tumor cells. **(A)** Regulation of cell surface expression of HLA-I molecules by EGF-mediated EGFR signaling was measured by flow cytometry. Cells were cultured in 1% FCS RPMI-1640 alone or supplemented with EGF (0.08 nM) during 48 h. **(B,C)** HLA-ABC, HLA-A, or HLA-B surface expression was assessed by flow cytometry in EGFRI-treated cells. Cells were treated with AG1478 (5 μM), nimotuzumab or cetuximab (10 μg/ml) in 1% FCS, 0.08 nM EGF RPMI-1640 medium during 96 h. Cells cultured in the presence of human IFN-γ (800 UI/ml) during 48 h were used as control of maximum HLA-I induction. Basal HLA-I expression was determined in untreated cells. For **(A–C)** bars represent a mean of HLA-I Δ MFI = MFI (staining with antibodies specific for HLA-I antigens) – MFI (staining with isotype control) values ± SD of three-four independent experiments. For **(B)**, a media of HLA-ABC increase index = HLA-ABC ΔMFI (treated-cells)/HLA-ABC ΔMFI (untreated-cells) values is included for each experimental group. For **(A)**, statistical analyses were performed using Mann–Whitney *U*-test (^∗^*P* < 0.05). For **(B,C)**, analyses were performed according to Kruskal–Wallis test and Dunn post-test (different letters indicate statistical differences).

We determined whether nimotuzumab mediates upregulation of HLA class I molecules and APM components in tumors. Previous results from Ferris and colleagues have suggested a dependence on EGFR density for cetuximab-mediated HLA class I modulation (Srivastava et al., 2015). Consequently, we selected two human tumor cell lines having high to intermedium EGFR expression levels, A431 that express 2–3 × 10^6^ EGFR molecules per cell (Haigler et al., 1978) and H125 that express 2.1 × 10^5^ EGFR molecules per cell (Suarez Pestana et al., 1997). U1906 cell line which does not express the EGFR at all (Suarez Pestana et al., 1997) was used as negative control. First, we examined how EGFR signaling impacts HLA class I membrane expression in these tumor cell lines. Previous findings showed that A431 and H125 cells have a maximum increase of cell viability after stimulation with 0.08 nM of EGF (Garrido et al., 2011b). EGF-mediated EGFR activation reduced significantly the expression levels of HLA-ABC molecules in A431 and H125 cells when compared with cells cultured in the absence of EGF (**Figure 1A**). A ≈4-fold decrease of HLA class I molecules expression was obtained for both EGF-cultured tumors. As expected, this effect was not confirmed in EGF-treated U1906 cells (**Figure 1A**). Subsequently, we explored the effect of nimotuzumab on HLA class I membrane expression, and included others EGFR inhibitors (cetuximab and AG1478) and IFN-γ as positive controls. Nimotuzumab at 10 μg/ml provoking a suboptimal reduction in cell viability for A431 and H125 cells (**Supplementary Figure S1A**), produced a significant increase in HLA-ABC membrane expression (**Figure 1B**). A similar HLA-I enhancement was achieved in cetuximab and AG1478-treated A431 and H125 tumor cells when compared with untreated cells. Notably, in EGFR inhibitor-treated cells, we found an increase index for HLA class I expression comparable to that produced by IFN-γ treatment. As expected, we did not detect increase in HLA class I expression in EGFR-inhibitor-treated U1906 cells (**Figure 1B**). In experiments with A431 and H125 cells we observed a dose-dependent effect of nimotuzumab on upregulation of HLA-ABC (**Supplementary Figure S1A**) with a maximum effect at 96 h of incubation (**Supplementary Figure S1B**). When we evaluated the effect of nimotuzumab on HLA-I locus-specific expression, an increase of membrane levels of HLA-A and HLA-B was verified in both EGFR-positive tumors when compared to untreated cells (**Figure 1C**). For A431 cells a similar increase was detected for both alleles, whereas for H125 cells upregulation of HLA-A alleles was more pronounced than HLA-B alleles.

**FIGURE 2:**
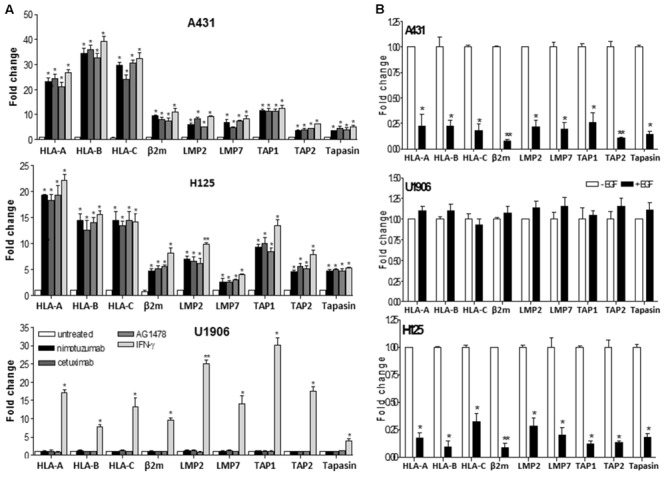
Nimotuzumab regulates the expression of HLA-I antigens, β_2_-m and APM components in human tumor cells on a transcriptional level. **(A)** Cells were treated with nimotuzumab or cetuximab (10 μg/ml, 96 h), AG1478 (5 μM, 96 h) or human IFN-γ (800 UI/ml, 48 h) in 1% FCS, 0.08 nM EGF RPMI-1640 medium. **(B)** Cells were cultured during 48 h in 1% FCS, 0.08 nM EGF RPMI-1640 medium. mRNA expression of HLA-I antigens, β_2_-m and APM components was examined by real-time qPCR analyses. Results from real-time qPCR are depicted as fold change from untreated cells **(A)** or cells cultured without EGF **(B)** (both cell conditions were set to 1) and assessed by the comparative threshold cycle method normalized to reference gene expression (GADPH). Data represent the mean ± SD of three independent experiments performed in quadruplicate. Analyses were performed using one-way ANOVA test and Dunnet post-test for untreated versus treated-cells comparisons **(A)** and using paired two-tailed Student’s *t*-test **(B)**. Statistical differences are indicated: ^∗∗^*P* < 0.01, ^∗^*P* < 0.05.

Next, we analyzed the effect of nimotuzumab on HLA class I molecules at transcriptional levels in comparison with cetuximab and AG1478. Similar to these EGFR inhibitors, a significant upregulation of mRNA levels of HLA-A, HLA-B, and HLA-C was seen in nimotuzumab-treated A431 and H125 cells when compared to untreated cells (**Figure 2A**). In contrast, a transcriptional induction of HLA class I HC genes was only detected in IFN-γ-treated U1906 cells (**Figure 2A**). Subsequently, to determine whether nimotuzumab-induced EGFR inhibition influences other molecules of HLA class I and APM machinery, we expanded our real-time quantitative PCR (qPCR) analyses to β_2_-microglobulin (β_2_-m), the complex for peptide generation/transport and the chaperones necessary for MHC-I assembly (**Figure 2A**). A significant increase in mRNA levels for β_2_-m, LMP subunits, TAPs, as well as tapasin was demonstrated in nimotuzumab-treated A431 and H125 cells when compared to untreated cells. Similar effects were induced by cetuximab and AG1478 in the selected tumor cells. In agreement with our previous findings, a relevant reduction of mRNA levels for HLA class I HC, β_2_-m and APM components was found in EGF-treated A431 and H125 cells when compared with cells cultured in the absence of this growth factor (**Figure 2B**). Addition of the EGF to U1906 cells did not reduce the levels of mRNA for HLA class I HC, β_2_-m and APM components (**Figure 2B**).

### *In Vivo* Treatment with 7A7 Increases the Surface Expression of MHC-I Molecules in D122 Tumors

**FIGURE 3:**
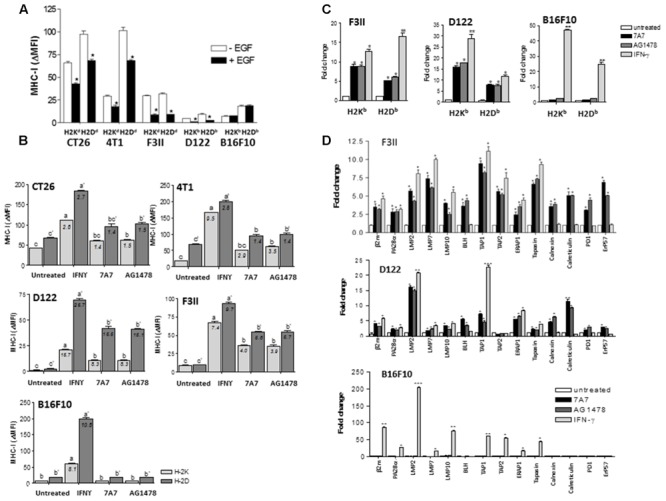
Blockade of EGF-mediated EGFR signals by 7A7 treatment enhances the mRNA levels of MHC-I HC, β_2_-m and APM genes in D122 and F3II tumor cells. **(A)** Regulation of MHC-I molecules surface expression by EGF-mediated EGFR signaling was measured by flow cytometry analysis. Cells were cultured in 1% FCS RPMI-1640 medium alone or supplemented with EGF (0.5 nM) during 48 h. **(B)** MHC-I surface expression was assessed by flow cytometry in 7A7-treated cells. Cells were treated with 7A7 (1 μg/ml) or AG1478 (5 μM) in 1% FCS, 0.5 nM EGF RPMI-1640 medium during 96 h. Cells cultured in the presence of mouse IFN-γ (100 UI/ml) during 48 h were used as control of maximum MHC-I induction. Basal MHC-I expression was determined in untreated cells. For **(A,B)**, bars represent a mean of MHC-I Δ MFI = MFI (staining with antibodies specific for MHC-I alleles) – MFI (staining with isotype control) values ± SD of three-five independent experiments. For **(A)**, analyses were performed using Mann–Whitney *U*-test (^∗^*P* < 0.05). For **(B)**, a media of MHC-I increase index = MHC-I DMFI (treated cells)/MHC-I DMFI (untreated cells) values is included for each experimental group analyses were performed according to Kruskal–Wallis test and Dunn post-test (different letters indicate statistical differences). **(C,D)** mRNA expression of MHC-I HC, β_2_-m and APM components was examined by real-time qPCR analyses. Cells were treated with 7A7 (1 μg/ml, 96 h), AG1478 (5 μM, 96 h) or mouse IFN-γ (100 UI/ml, 48 h) in 1% FCS, 0.5 nM EGF RPMI-1640 medium. Results from real-time qPCR are depicted as described in **Figure 2**. Data represent the mean ± SD of three independent experiments performed in quadruplicate. For **(C,D)**, analyses were performed using one-way ANOVA test and Dunnet post-test for untreated versus treated-cells comparisons. Statistical differences are indicated: ^∗∗∗^*P* < 0.001, ^∗∗^*P* < 0.01, ^∗^*P* < 0.05.

Examples of anti-EGFR antibodies augmenting HLA class I expression in tumors mostly come from *in vitro* studies. In order to combine the preclinical evidence using immunocompetent mice, we used 7A7 a surrogate antibody that recognizes murine EGFR. In these experiments, the effect of AG1478 on MHC-I expression was also determined. We selected four EGFR-positive murine tumors from different histological origins (CT26, colon; 4T1 and F3II, mama; D122, lung) and included melanoma B16F10 cells as negative control for EGFR expression. Results from flow cytometry showed that F3II and D122 cells have similar membrane levels of EGFR which were ≈2-fold higher than in CT26 and 4T1 cells with comparable EGFR membrane levels (**Supplementary Figure S2A**). Using 0.5 nM of EGF, a concentration that induces an increase in cell viability for all EGFR-positive cells (**Supplementary Figure S2B**), a significant reduction in MHC-I expression levels was achieved for these tumors as compared to cells cultured without EGF (**Figure 3A**). It is noteworthy that MHC-I repression was slightly affected by EGFR expression levels. A ≈3.5-fold decrease of H-2K and H-2D expression was detected for F3II and D122 cells, whereas MHC-I HC reduction for CT26 and 4T1 cells was of ≈1.5-fold. EGF presence in culture medium did not affect MHC-I surface expression for B16F10 cells (**Figure 3A**). As expected, EGFR inhibition by 7A7, even in suboptimal cell viability reducing concentration (7A7: 1 μg/ml, **Supplementary Figure S2C**), had an opposite effect on MHC-I membrane expression in EGFR-positive tumors (**Figure 3B**). Treatment with 7A7 caused upregulation of MHC-I expression, effect which also depended on EGFR expression levels. In tumor cells with higher EGFR expression (F3II and D122), we observed a strong MHC-I upregulation after 7A7 treatment. In contrast, MHC-I induction mediated by 7A7 in CT26 and 4T1 cells with lower EGFR density, was less potent. We did not detect MHC-I increase in EGFRIs-treated B16F10 cells- in which only the IFN-γ treatment was able to induce MHC-I increase. For all EGFR-positive tumors, the greatest H-2D and H-2K induction by EGFR inhibitors treatment was detected at 96 h (**Supplementary Figure S2D**), and was dose dependent (**Supplementary Figure S2C**).

To determine whether the results obtained with human tumor cell lines could be reproduced in murine tumors, we conducted transcriptional studies using F3II and D122 tumor cells (mouse cells with higher EGFR expression). Similar to IFN-γ-cultured cells, a significant augment in mRNA levels for H-2K and H-2D was detected in 7A7 and AG1478-treated F3II and D122 cells when compared with untreated cells (**Figure 3C**). In contrast, a transcriptional induction of MHC-I HC genes was only detected in IFN-γ-treated B16F10 cells (**Figure 3C**). In addition, a significant increase in mRNA levels for β_2_-m, LMP subunits, PA28α, BLH, TAPs, ERAAP1, tapasin, calnexin, calreticulin, PDI, as well as ERp57, was found in EGFRI-treated F3II and D122 cells when compared with untreated cells. Of note, EGFR blockade changed the expression of several genes also inducible by IFN-γ, but some genes were exclusively influenced by EGFRIs (BLH, calnexin, calreticulin, PDI, and ERp57) (**Figure 3D**). In agreement with our previous findings, a relevant reduction of mRNA levels for MHC-I HC, β_2_-m and APM components was found in EGF-treated F3II and D122 cells when compared to cells cultured in absence of this growth factor (**Supplementary Figures S3A,B**). EGF addition to B16F10 cells did not decrease the levels of mRNA for MHC-I APM (**Supplementary Figure S3C**).

**FIGURE 4:**
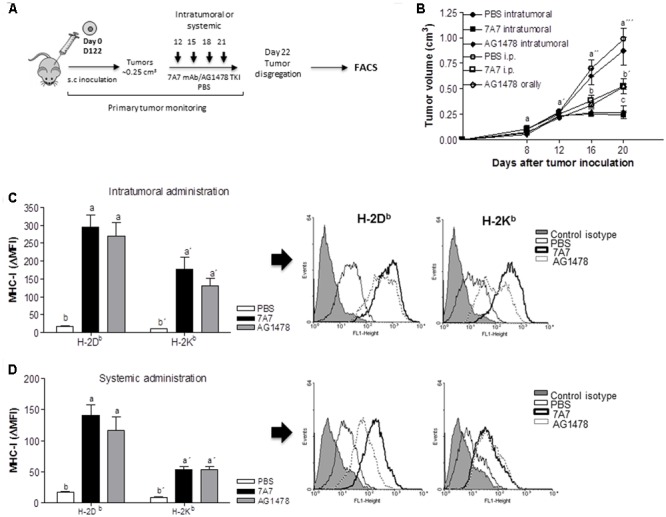
*In vivo* treatment with 7A7 enhances MHC-I expression in D122 tumors. **(A)** Schematic representation of D122 primary tumor model. Mice were challenged subcutaneously with D122 (0.5 × 10^6^) cells. Intratumoral or systemic administration schedule with 7A7 (56 μg), AG1478 (1 mg) or PBS began on day 12 when tumors reached ∼0.25 cm^3^ and continued as shown. **(B)** Primary tumor growth was monitored. One representative experiment out of two performed experiments is shown (*n* = 8). Each point represents mean ± SD of tumor volume in each group of mice. **(C,D)** D122 tumors were removed on day 22, MHC-I surface expression was measured by flow cytometry analyses in primary tumor-derived cell suspensions. Bars represent a mean of MHC-I Δ MFI = MFI (staining with antibodies specific for MHC-I alleles) – MFI (staining with isotype control) values ± SD of eight independent mice. Representative flow cytometry pictograms are shown. Analyses were performed according to two-way ANOVA test and Bonferroni post-test **(B)** and to Kruskal–Wallis test and Dunn post-test **(C,D)**. Different letters indicate statistical differences.

Next, we studied the capacity of 7A7 to alter the MHC-I expression *in vivo*. To conduct these experiments we selected D122 cells since they demonstrated a higher MHC-I induction after treatment with EGFRIs as compared to other EGFR-positive tumor cells (**Figure 3B**). Moreover, our previous experiments showed that 7A7 and AG1478, after 21 days of tumor inoculation, decrease both primary tumor as well as the appearance of experimental and spontaneous D122 metastases (Garrido et al., 2007, 2011a). D122 tumor-bearing mice were treated with 7A7 or AG1478 in two modalities as described in **Figure 4A**. Consistent with our previous studies, administration of 7A7 and AG1478 significantly reduced tumor volume when compared to control mice (**Figure 4B**). As expected, animals receiving intratumoral injections of anti-EGFR agents achieved a higher antitumor effect than those injected systemically. MHC-I surface expression was studied by flow cytometry of cells isolated from primary tumors. Intratumoral inoculation of 7A7 and AG1478 promoted a strong induction of H-2K^b^ and H-2D^b^ expression when compared with control mice (**Figure 4C**). Notably, this effect was also achieved in systemic studies (**Figure 4D**). The effect of 7A7 antibody administration on MHC cell surface tumor expression was even higher than in case of AG1478 administration.

### Treatment with 7A7 Enhances the Susceptibility of D122 Tumor to CD8^+^ T Cell-Mediated Lysis

**FIGURE 5:**
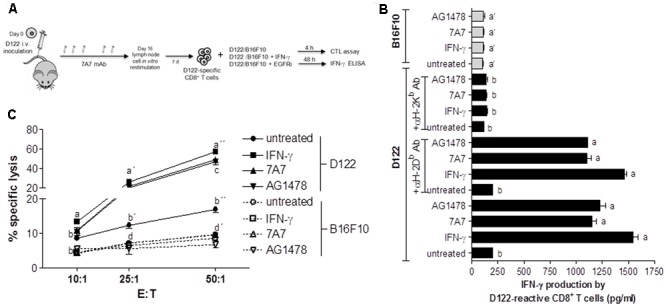
7A7 augments antitumor response of D122-specific CD8^+^ T cells. **(A)** D122-specific CD8^+^ T cells were generated in D122 experimental metastasis-bearing mice receiving 7A7 treatment as illustrated. **(B)** D122-reactive CD8^+^ T cells were incubated with D122 or B16F10 cells [untreated, treated with 7A7 (1 μg/ml, 96 h), AG1478 (5 μM, 96 h), or mouse IFN-γ (100 UI/ml, 48 h)] in a ratio 2.5:1, 48 h later IFN-γ secretion was quantified by ELISA. D122 recognition by tumor-specific CD8^+^ T cells was blocked by addition of anti-H-2K^b^ or anti-H-2D^b^ antibodies (10 μg/ml), and its effect on IFN-γ production was also determined. Bars represent mean of triplicate measurements ± SD. **(C)** Cytotoxic activity was determined by *in vitro* lactate dehydrogenase assay using target cells described above. Percentage of specific lysis was calculated as: [(experimental release – effector cell release – spontaneous release)/(maximum release – spontaneous release)] × 100. Each point represents mean of triplicate measurements ± SD. One representative experiment out of two performed experiments is shown in each case. Statistical analyses were performed according to Kruskal–Wallis test and Dunn post-test **(B)** and using two-way ANOVA test and Bonferroni post-test **(C)**. Different letters indicate statistical differences.

We also investigated whether MHC-I increase induced by 7A7 treatment in D122 cells leads to enhanced antitumor immunity. To this end, the ability of D122-reactive CD8^+^ T cells to secrete IFN-γ in response to tumor recognition was studied by co-incubation of these T cells with D122 pretreated or not with 7A7 or AG1478 (**Figure 5A**). As shown in **Figure 5B**, D122 treatment with EGFRIs increased significantly the IFN-γ production by D122-specific CD8^+^ T cells when compared to untreated cells. Remarkably, the concentrations of IFN-γ detected in supernatants from CD8^+^ T cells: EGFRI-treated D122 co-cultures were similar to those obtained in CD8^+^ T cells: IFN-γ-treated D122 co-cultures. B16F10 cells (that expressed matching MHC-I but did not present appropriate antigens) failed to stimulate D122-reactive CD8^+^ T cells to release IFN-γ (**Figure 5B**). Consistent with a predominant role for H-2K^b^ molecules in the T cell-based cytotoxic response to D122 tumor (Eisenbach et al., 1984; Mandelboim et al., 1992; Plaksin et al., 1992), the capacity of 7A7 and AG1478-treated D122 to stimulate D122-reactive CD8^+^ T cells was strongly blocked by the addition of an anti-H-2K^b^ antibody, whereas the presence in the culture of an anti-H-2D^b^ antibody did not reduce the tumor recognition (**Figure 5B**). Finally, cytotoxic activity of D122-specific CD8^+^ T cells co-cultured with EGFRIs-treated D122 cells was examined (**Figure 5A**). Our results demonstrated that 7A7 and AG1478 treatment of D122 cells enhance their susceptibility to be lysed by CD8^+^ T cells (**Figure 5C**). T cells eliminated a high percentage of EGFRIs-treated D122 cells (∼48% using an E:T ratio of 50:1). This response was not present when B16F10 cells were used as target cells (**Figure 5C**).

## Discussion

Today, EGFR-targeting therapies constitute an increasingly important strategy in the clinical management of tumors. Although these immunotherapeutic agents provide a clinical benefit for advanced cancer patients, the actual advantage in terms of median overall survival is rather limited due tumor escape mechanisms. Combinations of EGFR-targeting therapies with immunomodulatory approaches and cancer vaccines constitute a promising way to increase their clinical effectiveness. Nevertheless, to optimally design these clinical trials, it is critical to understand how EGFRIs influence the expression of genes coding for immune regulators, especially those that govern the interaction between tumors and T cells (for example, HLA class I molecules). In the last 10 years, many groups have explored the influence of anti-EGFR agents on HLA class I expression in tumors and found that TKIs (such as PD168393, AG1478 and gefinitib), as well as the antibody cetuximab, enhance HLA class I expression (Kersh et al., 2016). Our current results showed that nimotuzumab also increases the tumor surface expression of HLA class I molecules. Several studies suggested that not always the blockade of HLA class I-related oncogenes results in HLA class I modulation. For example, neither HER-2 blockade by the mAb Herceptin (Kono et al., 2004) nor RAS pathway interference with vemurafenib (a BRAF inhibitor) produced an increase in HLA class I surface expression (Boni et al., 2010; Koya et al., 2012). In agreement with the results obtained by Ferris and colleagues using cetuximab (Srivastava et al., 2015), we showed that nimotuzumab augments HLA class I HC, β_2_-m and APM components at transcriptional level. Similarly, we found that nimotuzumab differentially enhances expression of HLA class I alleles. Additional work is needed to measure changes in the HLA class I expression using tumor biopsies taken from cancer patients during nimotuzumab therapy to demonstrate how it affects patient survival. In this context, cetuximab neoadjuvant therapy has demonstrated to upregulate HLA class I expression in tumors of responder patients but not in non-responders (Srivastava et al., 2015).

Taking into account that nimotuzumab recognize the human EGFR but not the murine counterpart, we used a preclinical animal model to study the possible modulation of MHC-I expression *in vivo* by nimotuzumab and its impact on antitumor immune response. Previously, we generated a surrogate antibody 7A7 that recognizes murine EGFR. Our studies based on immunocompetent mice (a complete autologous setting) demonstrated that 7A7 manifests antitumor effects via mechanisms that are identical to those attributed to mAbs targeting human EGFR, namely, (1) EGFR signaling inhibition, (2) antibody dependent cell-mediated cytotoxicity, and (3) T cell activation (Garrido et al., 2007, 2011b). Collectively, these results validated 7A7 as a valuable preclinical tool. An association between MHC-I alterations and EGFR-targeting antibody-induced resistance was recently described using 7A7 (Garrido et al., 2013, 2014) supporting the increase in MHC-I expression as a relevant antitumor mechanism of EGFR blocking antibodies. We demonstrated that 7A7 increases tumor cell surface expression of MHC-I *in vivo*, and we also showed that this antibody increases tumor susceptibility to CD8^+^ T cells-mediated lysis. In this experimental model, we also extended the characterization of APM components regulated by EGFRIs and found that 7A7 regulates MHC-I surface expression via a coordinated effect on mRNA levels of several genes related to this pathway. Our results demonstrated that EGFR blockade modulates the expression of several genes regulated by IFN-γ, suggesting that EGFRIs and IFN-γ could use similar signaling pathways and transcription factors to increase MHC-I expression. In this context, it has been recently described that EGFR-SHP2-STAT1 pathway regulates HLA class I expression in tumors due EGFR inhibition by cetuximab enhanced IFNγ receptor 1 expression, augmenting induction of HLA class I expression by IFNγ, which was abrogated in STAT1-/- cells (Srivastava et al., 2015). However, we also observed genes exclusively influenced by EGFR-inhibitors; hence, the contribution of additional signaling pathways cannot be ruled out. Experimental observations from Sers et al. (2009) demonstrated that U0126 (MEK inhibitor) is able to regulate HLA class I expression. Also, studies of Mimura et al. (2013) demonstrated there exists a strong inverse correlation between p-Erk expression and HLA class I expression in clinical tumor, samples from gastric and esophageal cancer patients, showing that the predominant regulator of HLA-I expression is the MAPK pathway. Interestingly, it has been reported that SHP2 activation by EGFR also promotes MAPK signaling (Agazie and Hayman, 2003). Thus, SHP2 could be considered as a pivotal molecular mediator in EGFR and HLA class I connection (Concha-Benavente et al., 2013). In regards to transcriptional factors, Pollack’s group suggested that EGFR blockade increase HLA class I expression through a mechanism that involves the class II transactivator protein (CIITA) (Pollack et al., 2011). CIITA has been also described as an IFN-γ-responsive protein that regulates HLA class I expression (Martin et al., 1997). Nonetheless, the involvement of other molecules traditionally associated with the transcriptional regulation of HLA class I antigen presentation pathway, such as Sp1, CREB, the nuclear factor (NF)-κB, E2F, p300 (Seliger, 2008), NLRC5 (Meissner et al., 2010) and Fhit (Romero et al., 2012) has not been described yet. Also, epigenetic mechanisms could be involved in the regulation of HLA expression by EGFR. In this regard, it has been recently described that EGFR activation increases miR-21 expression, which has anti-inflammatory effects (Prabowo et al., 2015).

In summary, we demonstrated a direct modulation of HLA class I expression by nimotuzumab in tumors and broadened our understanding of how EGFR-targeting therapies may affect antitumor immune response. Discovery of this novel immune-related antitumor mechanism of nimotuzumab can help to monitor the combinatorial regimen-based clinical trials and makes nimotuzumab an attractive therapy for tumors carrying reversible defects (“soft lesions”) (Garrido et al., 2010a,b) in HLA class I expression.

## Author Contributions

Each author has contributed significantly to the submitted research. GG, AR, CG, LC designed and performed experiments and analyze data. GG wrote the paper. FG, AG-L, BS-R designed experiments, analyzed data and made critical revision of the manuscript.

## Conflict of Interest Statement

The authors declare that the research was conducted in the absence of any commercial or financial relationships that could be construed as a potential conflict of interest.

## Supplementary Material

FIGURE S1 Cells were treated with different concentrations of nimotuzumab, cetuximab or AG1478 during 96 h in 1% FCS, 0.08 nM EGF RPMI-1640 medium. HLA-I surface expression was measured by flow cytometry. Each point represents a mean of HLA-ABC ΔMFI = MFI (staining with antibodies specific for HLA-ABC) – MFI (staining with isotype control) values ± SD of three independent experiments. *In vitro* sensitivity to EGFRI treatment was determined by MTT assay. Untreated cells were included as maximum cell viability. Percentage of viable cells was determined as: (AbsΔ 540–620 nm of treated cells/AbsΔ 540–620 nm of untreated cells) × 100. Each point represents mean of triplicate measurements ± SD. **(B)** HLA-I surface expression was assessed by flow cytometry analyses in EGFRi-treated cells. Cells were treated with anti-EGFR antibodies (10 μg/ml) or AG1478 (5 μM) in 1% FCS, 0.08 nM EGF RPMI-1640 medium during 12, 24, 48, 72, 96, and 120 h. Basal MHC-I expression was determined in untreated cells. Each point represents a mean of HLA-ABC Δ MFI = MFI (staining with antibodies specific for HLA-ABC) – MFI (staining with isotype control) values ± SD of three independent experiments.

FIGURE S2 **(A)** EGFR surface expression was measured by flow cytometry analyses. Bars represent a mean of EGFR Δ MFI (mean fluorescence intensity) = MFI (staining with 7A7) – MFI (staining with isotype control) values ± SD of three independent experiments. **(B)** Cells were treated with different concentrations of EGF for 48 h. Cell survival, expressed as Abs 540–620 nm, was measured by MTT assay. Each point represents mean of triplicate measurements ± SD. **(C)** Cells were treated with different concentrations of 7A7 or AG1478 during 96 h in 1% FCS, 0.5 nM EGF RPMI-1640 medium. MHC-I surface expression was measured by flow cytometry analyses. Each point represents a mean of MHC-I Δ MFI = MFI (staining with antibodies specific for MHC-I alleles) – MFI (staining with isotype control) values ± SD of three independent experiments. *In vitro* sensitivity to EGFRI treatment was determined by MTT assay. Untreated cells were included as maximum cell viability. Percentage of viable cells was determined as: (AbsΔ 540–620 nm of treated cells/Abs Δ 540–620 nm of untreated cells) × 100. Each point represents mean of triplicate measurements ± SD. **(D)** MHC-I surface expression was assessed by flow cytometry analyses in EGFR inhibitors-treated cells. Cells were treated with 7A7 (1 μg/ml) or AG1478 (5 μM) in 1% FCS, 0.5 nM EGF RPMI-1640 medium during 12, 24, 48, 72, 96, and 120 h. Basal MHC-I expression was determined in untreated cells. Each point represents a mean of MHC-I Δ MFI values ± SD of three independent experiments.

FIGURE S3 D122 **(A)**, F3II **(B)**, and B16F10 **(C)** cells were cultured during 48 h in 1% FCS, 0.5 nM EGF RPMI-1640 medium. mRNA expression of MHC-I HC, β2-m and APM components was examined by real-time qPCR analyses. Results from real-time qPCR are depicted as fold change from cells culture without EGF and assessed by the comparative threshold cycle method normalized to reference gene expression (GADPH). Data represent the mean ± SD of three independent experiments performed in quadruplicate. Analyses were performed using paired two-tailed Student’s *t*-test. Statistical differences are indicated: ^∗^*P* < 0.05.

## Abbreviations

APM: antigen processing machinery
β_2_-m: β_2_-microglobulin
EGFR: epidermal growth factor receptor
HCs: heavy chains
mAb: monoclonal antibody
MFI: mean fluorescence intensity
TKI: tyrosine kinase inhibitor
