# Supplementary figures and images for: Upregulation of HLA Class I Expression on Tumor Cells by the Anti-EGFR Antibody Nimotuzumab
Source: Front Pharmacol. 2017 Oct 6;8:595. doi: 10.3389/fphar.2017.00595 (PMC5635422; doi:10.3389/fphar.2017.00595)

**A**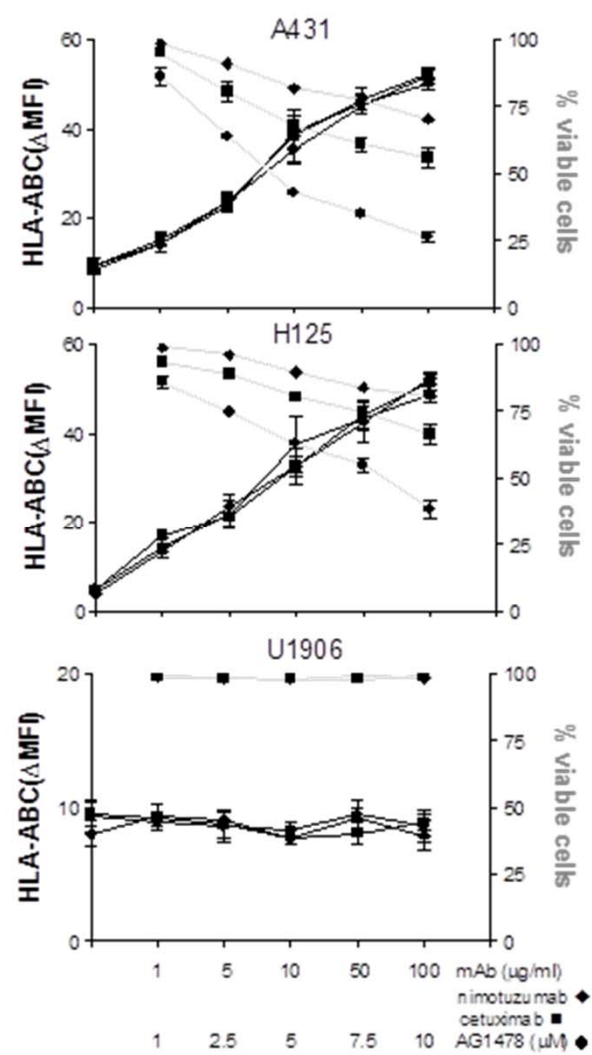**B**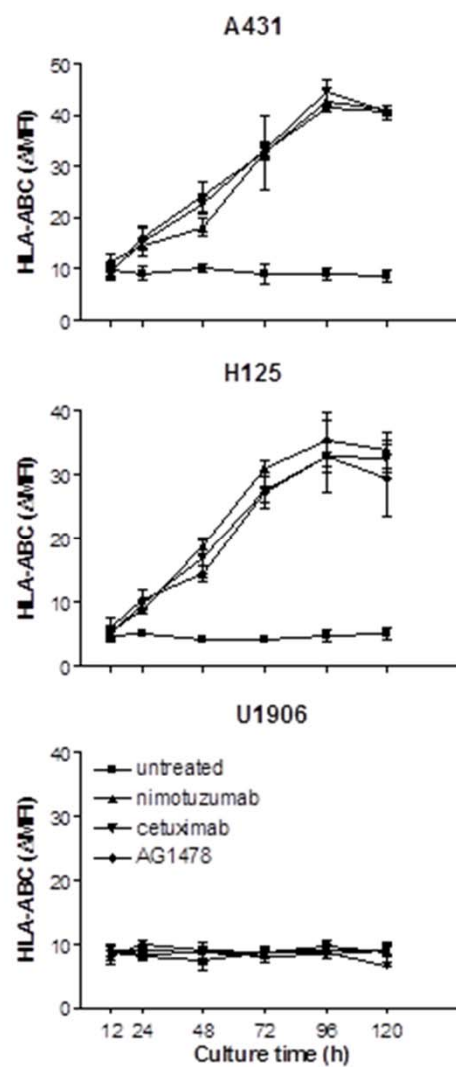

Supplement: FIGURE S1 — Cells were treated with different concentrations of nimotuzumab, cetuximab or AG1478 during 96 h in 1% FCS, 0.08 nM EGF RPMI-1640 medium. HLA-I surface expression was measured by flow cytometry. Each point represents a mean of HLA-ABC ΔMFI = MFI (staining with antibodies specific for HLA-ABC) – MFI (staining with isotype control) values ± SD of three independent experiments. In vitro sensitivity to EGFRI treatment was determined by MTT assay. Untreated cells were included as maximum cell viability. Percentage of viable cells was determined as: (AbsΔ 540–620 nm of treated cells/AbsΔ 540–620 nm of untreated cells) × 100. Each point represents mean of triplicate measurements ± SD. (B) HLA-I surface expression was assessed by flow cytometry analyses in EGFRi-treated cells. Cells were treated with anti-EGFR antibodies (10 μg/ml) or AG1478 (5 μM) in 1% FCS, 0.08 nM EGF RPMI-1640 medium during 12, 24, 48, 72, 96, and 120 h. Basal MHC-I expression was determined in untreated cells. Each point represents a mean of HLA-ABC Δ MFI = MFI (staining with antibodies specific for HLA-ABC) – MFI (staining with isotype control) values ± SD of three independent experiments. [file Presentation_1.PDF]

**A**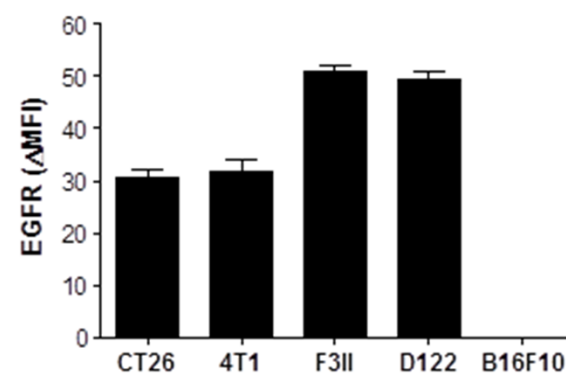**C**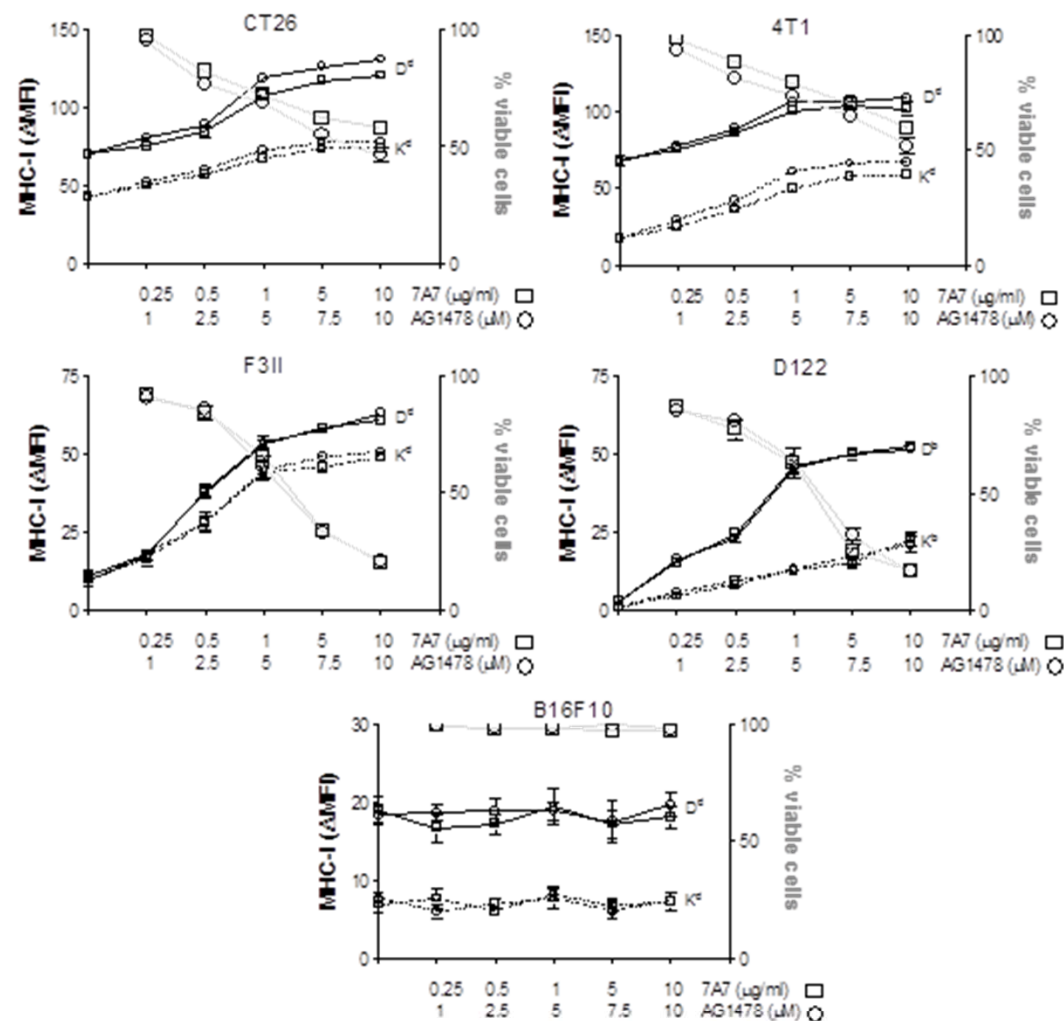**B**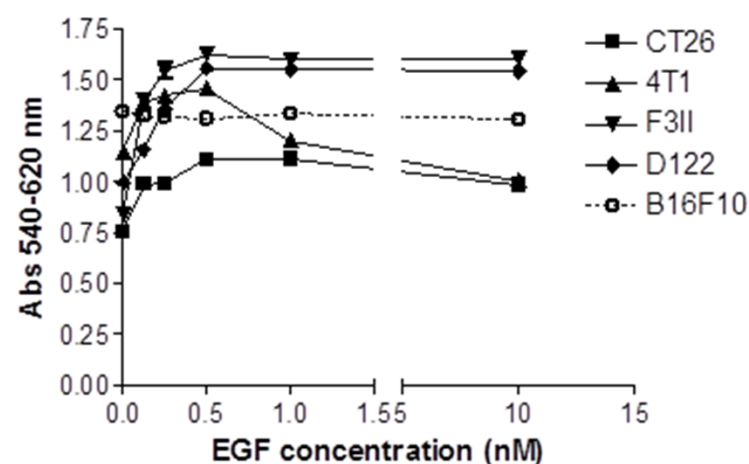**D**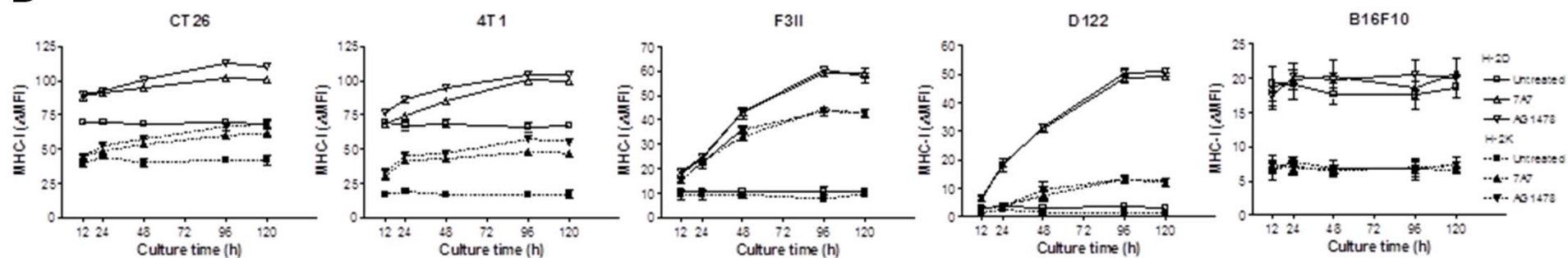

Supplement: FIGURE S2 — (A) EGFR surface expression was measured by flow cytometry analyses. Bars represent a mean of EGFR Δ MFI (mean fluorescence intensity) = MFI (staining with 7A7) – MFI (staining with isotype control) values ± SD of three independent experiments. (B) Cells were treated with different concentrations of EGF for 48 h. Cell survival, expressed as Abs 540–620 nm, was measured by MTT assay. Each point represents mean of triplicate measurements ± SD. (C) Cells were treated with different concentrations of 7A7 or AG1478 during 96 h in 1% FCS, 0.5 nM EGF RPMI-1640 medium. MHC-I surface expression was measured by flow cytometry analyses. Each point represents a mean of MHC-I Δ MFI = MFI (staining with antibodies specific for MHC-I alleles) – MFI (staining with isotype control) values ± SD of three independent experiments. In vitro sensitivity to EGFRI treatment was determined by MTT assay. Untreated cells were included as maximum cell viability. Percentage of viable cells was determined as: (AbsΔ 540–620 nm of treated cells/Abs Δ 540–620 nm of untreated cells) × 100. Each point represents mean of triplicate measurements ± SD. (D) MHC-I surface expression was assessed by flow cytometry analyses in EGFR inhibitors-treated cells. Cells were treated with 7A7 (1 μg/ml) or AG1478 (5 μM) in 1% FCS, 0.5 nM EGF RPMI-1640 medium during 12, 24, 48, 72, 96, and 120 h. Basal MHC-I expression was determined in untreated cells. Each point represents a mean of MHC-I Δ MFI values ± SD of three independent experiments. [file Presentation_2.PDF]

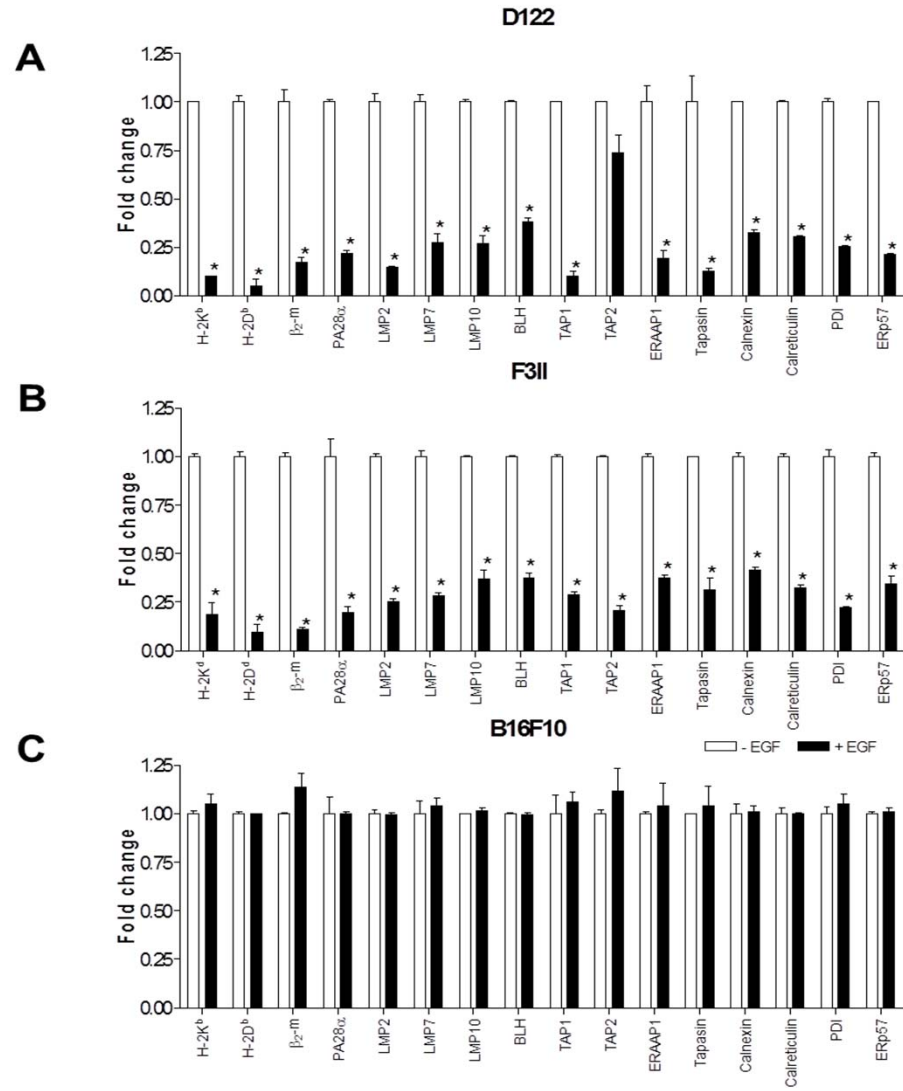

Supplement: FIGURE S3 — D122 (A), F3II (B), and B16F10 (C) cells were cultured during 48 h in 1% FCS, 0.5 nM EGF RPMI-1640 medium. mRNA expression of MHC-I HC, β2-m and APM components was examined by real-time qPCR analyses. Results from real-time qPCR are depicted as fold change from cells culture without EGF and assessed by the comparative threshold cycle method normalized to reference gene expression (GADPH). Data represent the mean ± SD of three independent experiments performed in quadruplicate. Analyses were performed using paired two-tailed Student’s t-test. Statistical differences are indicated: ∗P < 0.05. [file Presentation_3.PDF]
