# Supplementary material for: Upregulation of HLA Class I Expression on Tumor Cells by the Anti-EGFR Antibody Nimotuzumab
Source: Front Pharmacol. 2017 Oct 6;8:595. doi: 10.3389/fphar.2017.00595 (PMC5635422; doi:10.3389/fphar.2017.00595)
Supplement: Supplementary file 4 [file Presentation_4.PDF]

Table S1. Sequences of primers used in real-time qPCR analyses

Mouse genes

Gene	Oligo	Sequence 5'-3'
GADPH	Forward	TCAAGAAGGTGGTGAAGCAGG
	Reverse	CGATCGAAGGTGGAAGAGTGG
H-2K^b	Forward	TGGAGCTGCAATAGTCACTGG
	Reverse	CTGGGAGAGACAGATCAGAGG
H-2D^b	Forward	CTCCGTCCACTGACTCTTAC
	Reverse	CCACAGCTCCAATGATGGCC
H-2K^d	Forward	CCATCCACTGTCTCCAACACG
	Reverse	CCACCTGTGTTCCTTCTCATC
H-2D^d	Forward	GCCTCCTTCATCCACCAAGAC
	Reverse	CACAGCTCCAAGGATGACCAC
β_2-m	Forward	CGGTCGCTTCAGTCGTCAGC
	Reverse	TCTTCAGAGCATCATGATGC
PA28α	Forward	TGGTCACTACCTGGTTGCAGC
	Reverse	GTGTGAAGGTTGGTCATCAGC
LMP2	Forward	CCTCTGCACCAGCACATCTTC
	Reverse	CGTGTAGCTCCAGCTGGTAG
LMP7	Forward	GGACCTCAGTCCTGAAGAGG
	Reverse	CAACCGTCTTCCTTCATGTGG
LMP10	Forward	ACCTCAGCTCTACGAGGTGC
	Reverse	CTTCCACCAACAGCTCTTGC
BLH	Forward	TCACTGCTGTCTCAGAGAAGG
	Reverse	ACATGCTTCTTGTCCACCACC
TAP1	Forward	GCTGTTCAGGTCCTGCTCTC
	Reverse	CTCCTTGCTCTCCACTCAGTG
TAP2	Forward	AGGAGCCTGTGCTGTTCTCG
	Reverse	CTATGAAGTCGTCTGCACAGG

Gene	Oligo	Sequence 5'-3'
ERAAP1	Forward	TCCTTCTCAAGTTGCTGAAGG
	Reverse	CTCCTGAGCATCCTCTCTGAG
Tapasin	Forward	CAGCTACCTCCAGTCACTGC
	Reverse	CCTAGCACCTTGAGGAGTCC
Calnexin	Forward	GCAGCTGAAGAGCGTCCATGG
	Reverse	TCATCCTTCACATCTGGCTGG
Calreticulin	Forward	AGCAGATGAAGGACAAGCAGG
	Reverse	CCTCTCATCTTCTTCGTCCTC
PDI	Forward	GTGAGATCACCAAGGAGAAGC
	Reverse	GTGTGTCTTGATCTCACCTCC
Erp57	Forward	TCATGCAGGAGGAGTTCTCG
	Reverse	TGCTACCACAACCTTGACAGG

Human genes

Gene	Oligo	Sequence 5'-3'
GADPH	Forward	GGAAGGTGAAGGTCGGAGTC
	Reverse	GAAGATGGTGATGGGATTTCCA
HLA-A	Forward	CTCTTTGGAGCTGTGATCACT
	Reverse	GAAGGGCAGGAACAAMTCTTG
HLA-B	Forward	GTCCTAGCAGTTGTGGTCATC
	Reverse	TCAAGCTGTGAGAGACACATCA
HLA-C	Forward	TCCTGGCTGTCCTAGCTGTC
	Reverse	CAGGCTTTACAAGTGATGAGAG
β_2-m	Forward	TGACTTTGTCACAGCCCAAGAT
	Reverse	CAATCCAAATGCGGCATCTTCA
LMP2	Forward	ATGGGTTCTGATTCCCGAGTG
	Reverse	GCTTGGGCATCAGCAGCTGA
LMP7	Forward	CCTTCAAGTTCCAGCATGGAG
	Reverse	GCTGCACAGCCAGACATGGT
TAP1	Forward	GCCTCACTGACTGGATTCTAC
	Reverse	TCTCCCTGCAAGTGGCTGTG
TAP2	Forward	GGTCGTGTGATTGACATCCTG
	Reverse	TCAGCTCCCCTGTCTTAGTCT
Tapasin	Forward	TCCAGCCTCTTGCGACCACA
	Reverse	CTCAAGTCCAGCAGAGCATCT
